# Gastrodia Elata Bl Attenuates Methamphetamine-Induced Dopaminergic Toxicity *Via* Inhibiting Oxidative Burdens

**DOI:** 10.2174/157015911795016967

**Published:** 2011-03

**Authors:** E.-J Shin, J.-H Bach, T.-T. L Nguyen, X.-K. T Nguyen, B.-D Jung, K.-W Oh, M. J Kim, S.K Ko, C.G Jang, S. F Ali, H.-C Kim

**Affiliations:** 1 Neuropsychopharmacology and Toxicology Program, College of Pharmacy, Kangwon National University, Chunchon 200-701, South Korea; 2 School of Veterinary Medicine, Kangwon National University, Chunchon 200-701, South Korea; 3 College of Pharmacy, Core Research Institute, Chungbuk National University, Cheongju 361-763, South Korea; 4 Division of Bio-Resources Technology, Kangwon National University, Chunchon 200-701, South Korea; 5 Department of Oriental Medical Food & Nutrition, Semyung University, Jecheon 390-711, South Korea; 6 Department of Pharmacology, College of Pharmacy, Sungkyunkwan University, Suwon440-746, South Korea; 7 Neurochemistry Laboratory, Division of Neurotoxicology, National Center of Toxicological Research, Food and Drug Administration, Jefferson, AR 72079, USA

**Keywords:** *Gastrodia elata* Bl, methamphetamine, dopamine, oxidative stress.

## Abstract

It has been recognized that *Gastrodia elata* Bl (GE), an oriental herb medicine, ameliorates various neurological disorders, that GE modulates the monoaminergic and GABAergic systems, and that GE possess antioxidant activities. We examined whether GE affects methamphetamine (MA)-induced striatal dopaminergic toxicity in mice. Treatment with MA (7.5 mg/kg, i.p. × 4) resulted in significant decreases in behavioural activity (as shown by locomotor activity and rota rod performance), dopamine level, tyrosine hydroxylase (TH) activity, and TH protein expression (as evaluated by immunocytochemistry and western blot analysis). In addition, MA treatment showed significant increases in lipid peroxidation [as evaluated by 4-hydroxy-2-nonenal (4-HNE) expression and malondialdehyde formation], protein oxidation (as shown by protein carbonyl expression and its formation), and reactive oxygen species (ROS) formation. Treatment with GE significantly attenuates MA-induced behavioural and dopaminergic impairments, and oxidative stresses in a dose-dependent manner. Our results suggest that GE treatment shows anti-dopaminergic effects in response to MA insult *via*, at least in part, inhibiting oxidative stresses in the striatum of the mice.

## INTRODUCTION

*Gastrodia elata* Bl (GE) is a well-known herb agent that has been used to treat headache, paralysis, migraine, and other neurological disorders in oriental countries for centuries [1]. Prolonged GE treatment significantly increased dopamine (DA) concentration and decreased DA turnover in the striatum of the rats, suggesting that GE modulates DA system in the rats [2]. Recently, we filed a patent (Korean PCT10-2010-0000818; “pharmaceutical compositions containing GE extract useful for Parkinson’s disease”) to extend neuropharmacological indication of GE.

Earlier evidences suggest that GE exerts antioxidant activities, which are attributable to its components (i.e. hydroxybenzyl aclcohol, vanillyl alcohol, vanillin and hydroxybenzaldehyde), and that their antioxidant effect *in vitro* is more potent than that by melatonin [3].

Methamphetamine (MA) leads to long-lasting depletion of nigrostriatal DA [4, 5], which is associated with increased oxidative damage [4, 6-9]. It has been suggested that humans who abuse MA have an increased risk for the PD later in life [6, 10]. Thus, MA-induced dopaminergic toxicity has been considered to be one of the important models for PD [5, 11, 12].

The present study was designed to investigate the pharmacological activity of GE on dopaminergic neurons to MA toxicity in mice. We evaluated the effects of GE on the dopaminergic impairments and oxidative stresses [i.e., lipid peroxidation and protein oxidation, and reactive oxygen species (ROS)] induced by MA.

## METHODS

All animals were treated in accordance with the NIH *Guide for the care and use of laboratory animals* (NIH Publication No. 85-23, 1985; www.dels.nas.edu/ila). This study was performed in accordance with the Institute for Laboratory Animal Research (ILAR) guidelines for the care and use of laboratory animals. Male C57BL/6J mice (Bio Genomic Inc., Charles River Technology, Gapyung-Gun, Gyeonggi-Do, South Korea) weighing 25 ± 3 g were maintained on a 12 h:12 h light:dark cycle and fed *ad libitum*.

MA was obtained from Korea Food & Drug Administration (KFDA) and dissolved in 0.9% sterile saline. Methanol extract of GE was obtained from Samsung Herb Medicine, Co. (Chunchon, South Korea) and suspended in 0.5 % carboxymethylcellulose. All solutions were immediately prepared before use. Experimental schedules in detail are shown in Fig. (**1A**). 

Locomotor activity and rota-rod performance were measured as described previously [13]. Striatal dopamine, 3,4-dihydroxyphenylacetic acid (DOPAC), homovanilic acid (HVA), and TH activity were measured by HPLC-electrochemical detection [13, 14]. Dopamine turnover rate was calculated by the ratio of [(DOPAC + HVA)/ dopamine]. TH expression in the striatum was examined by western blot or immunocytochemical analyses [13] using primary antibody against TH (1:5,000, Chemicon, Temecula, MA, USA) or β-actin (1:50,000, Sigma-Aldrich, St Louis, MO, USA). Protein oxidation was measured by slot blot analysis [13] using Oxyblot kit (Chemicon, Temecula, MA, USA) or by spectrophotometrical determination using 2,4-dinitro-phenylhydrazine (DNPH)-labeling procedure [13]. Lipid peroxidation was determined by slot blot analysis using primary antibody against 4-hydroxy-2-nonenal (4-HNE) (Calbiochem, Gibbstown, NJ, USA) [13] or by HPLC-UV/VIS detection of malondialdehyde [13]. Synaptosomal reactive oxygen species (ROS) was measured as described previously [15]. Striatal synaptosomes from four animals pooled for each assay. Statistical analyses were performed using one-way analysis of variance (ANOVA) or repeated measure one-way ANOVA. A *post-hoc* Fisher’s PLSD test was then applied. A *p *value < 0.05 was deemed to indicate statistical significance.

## RESULTS AND DISCUSSION

**Fig. (1):**
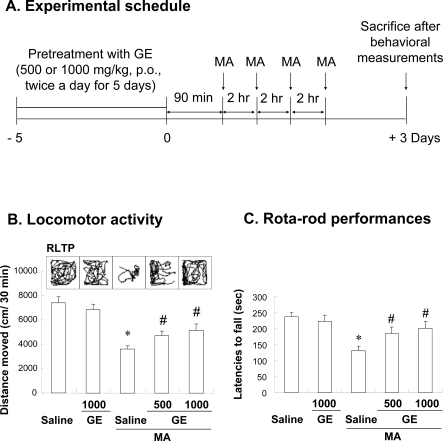
Experimental schedule for evaluating the effects of Gastrodia elata Bl (GE) on methamphetamine (MA)-induced dopaminergic toxicity (**A**), and effects of GE on the MA-induced behavioural impairments [locomotor activity (**B**) and rota rod performance (**C**)]. Note MA-induced behavioural impairment as shown by representative locomotor tracing patterns (RLTP). GE significantly attenuates this impairment. Mice received GE (500 or 1000 mg/kg, p.o.) twice a day for consecutive 6 days. Mice received first MA 90 min after final GE. Mice received four times of MA (7.5 mg/kg. i.p.) by 2 hours’ time schedule. Mice were sacrificed 3 days after final MA treatment to examine biochemical parameters. Each value is the mean ± S.E.M. of 10 animals. *p < 0.01 vs. Saline, ^#^*p* < 0.05 vs. Saline + MA (One-way ANOVA followed by Fischer’s PLSD test).

Experimental protocol for this study was shown in Fig. (**1A**). Mice receiving GE (500 or 1000 mg/kg, p.o.) alone did not show any specific behaviours. Three days after final MA treatment, mice showed significant decreased locomotor activity (*p* < 0.01 vs. Saline) and rota rod performance (*p* < 0.01 vs. Saline) (Fig. **1B** and **C**). Representative locomotor tracing patterns also support respective locomotor activity (Fig. **1B**). GE treatment attenuated these behavioural impairments (Locomotor activity and Rota rod performance; Saline + MA vs. GE 500 or 1000 mg/kg + MA, *p* < 0.05) (Fig. **1B** and **C**).

**Fig. (2):**
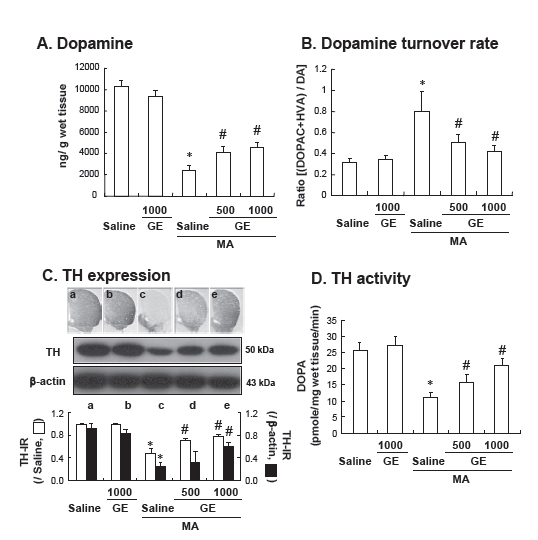
Effects of Gastrodia elata Bl (GE) on the striatal changes in dopamine level (**A**), dopamine turnover rate (**B**), tyrosine hydroxylase (TH) expression (as assessed by immunocytochemistry and western blot) (**C**) and TH activity (**D**) induced by methamphetamine (MA). Each value is the mean ± S.E.M. of 6 animals (for dopamine, dopamine turn over rate, and TH activity), and of 4 animals (for immunocytochemistry and western blot analysis). *p < 0.01 vs. Saline, ^#^*p* < 0.05 vs. Saline + MA (One-way ANOVA followed by Fischer’s PLSD test).

MA treatment significantly decreased dopamine levels (*p* < 0.01 vs. Saline), while dopamine turnover rate was significantly increased (*p* < 0.01 vs. Saline). GE treatment significantly attenuated changes in dopamine levels (Saline + MA vs. GE 500 or 1000 mg/kg + MA, *p* < 0.05) and dopamine turnover rate (Saline + MA vs. GE 500 mg/kg + MA or GE 1000mg/kg + MA, *p* < 0.05) and induced by MA (Fig. **2A** and **B**). 

In this study, changes in tyrosine hydroxylase (TH) activity are in line with those in TH expression (as shown by immunocytochemistry and western blot analysis). Consistently, tendency of TH activity and TH expressions apparently is in line with that of dopamine levels (Fig. **2C** and **D**). 

Fig. (**3**). showed the changes in the lipid peroxidation (as shown by 4-HNE expression and MDA level), protein oxidation (as shown by protein carbonyl expression and protein carbonyl level) and ROS. A mild expression of 4-HNE was observed in saline- or GE-treatment. MA significantly increased 4-HNE expression (*p* < 0.01 vs. Saline). The result in 4-HNE expression was consistent with that in MDA level. A mild to moderate expression of protein carbonyl was noted in the saline or GE-treated group. Similar to 4-HNE, MA treatment significantly increased (*p* < 0.01 vs. Saline) protein carbonyl expression and its level. In addition, formations of ROS are apparently in line with those of MDA and protein carbonyl. GE treatment was significantly inhibited these markers of oxidative stress [expressions of HNE and protein carbonyl, and formations of MDA, protein carbonyl and ROS: Sal + MA vs. GE (500 mg/kg or 1000 mg/kg) + MA, *p* < 0.05].

In the present study, we showed that GE significantly attenuates MA-induced impairments in the behavioural activity, dopaminergic function, and that GE significantly inhibited MA-induced pathologic oxidative changes. Therefore, our results indicate that GE-mediated antioxidant effects may contribute to block MA-induced dopaminergic toxicity in mice. To our knowledge, this is the first time that GE attenuates dopaminergic toxicity. 

Major components of GE are vanillyl alcohol, phenolic compounds, organic acids, glucose, beta-sitosterol, hydroxybenzyl alcohol, vanillin, gastrodin and hydroxybenzaldehyde [16]. Vanillyl alcohol showed antioxidant effects in response to excitotoxic condition in rats [17]. Gastrodin was reported to increase the GABA level by inhibiting the GABA shunt [18]. Recently, it was suggested that the order of antioxidant potency of components of GE is hydroxylbenzyl alcohol > vanillyl alcohol > vanillin > hydroxybenzaldehyde [3]. Correspondingly, Liu and Mori [19] demonstrated that GE inhibits cerebral lipid peroxidation *via* in part modulating antioxidant enzymes.

Many evidence indicated that GE mainly modulates GABAergic system, although GE also modulates dopaminergic and serotonergic systems. Earlier study demonstrated that high-dose MA enhances dopamine D1 receptor-mediated nigrostriatal GABAergic transmission, which activates GABA_A_ receptors in the substantia nigra, leading to a decrease in GABAergic nigrothalamic activity, and increase in corticostriatal glutamate release, and a consequent long-term depeletion of striatal dopamine contents [20]. This finding may be associated with increased formation of free radical [7]. This increase in free radical formation consequently results in increases in markers of lipid peroxidation and protein oxidation, namely MDA/4HNE and protein carbonyl as shown in this study. Thus, we raise the possibility that GABAergic effects of GE may be, at least in part, important for GE-mediated neuroprotection. We observed in our pilot study that anti-dopaminergic effects of GE are more pronounced than those of respective component of GE and the dose less than 500 mg/kg GE is not effective in preventing MA toxicity (data not shown). In addition, it remains to be fully determined on the interaction between GABAergic, dopaminergic populations and antioxidant components after exposed to GE. Combined, we indicate that antioxidant properties of GE may be helpful for treatment of oxidative stress responsible neurological disorders, although it’s precise mechanism remains elusive.

## Figures and Tables

**Fig. (3):**
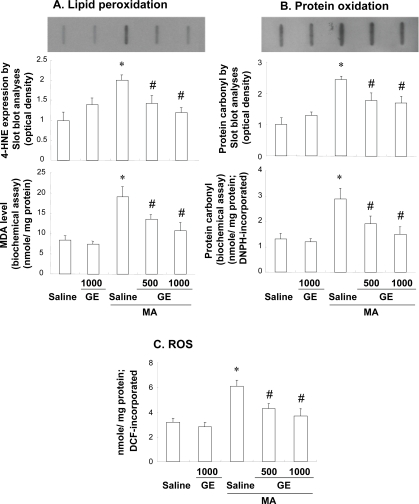
Effects of Gastrodia elata Bl (GE) on the lipid peroxidation [as shown by 4-hydroxy-2-nonenal (4-HNE) expression and malondialdehyde (MDA) level] (**A**), protein oxidation [as assessed by protein carbonyl expression and its level] (**B**), and reactive oxygen species (ROS) induced by methamphetamine (MA). Each value is the mean ±S.E.M. of 6 animals. *p < 0.01 vs. Saline, ^#^*p* < 0.05 vs. Saline + MA (One-way ANOVA followed by Fischer’s PLSD test).
